# Extracellular Vesicle Therapy for Type 1 Diabetes

**DOI:** 10.3389/fimmu.2022.865782

**Published:** 2022-04-08

**Authors:** Setareh Soltani, Kamran Mansouri, Mohammad Sajad Emami Aleagha, Narges Moasefi, Niloofar Yavari, Seyed Kazem Shakouri, Sara Notararigo, Ali Shojaeian, Flemming Pociot, Reza Yarani

**Affiliations:** ^1^Clinical Research Development Center, Taleghani and Imam Ali Hospital, Kermanshah University of Medical Sciences, Kermanshah, Iran; ^2^Medical Biology Research Center, Health Technology Institute, University of Medical Sciences, Kermanshah, Iran; ^3^Medical Technology Research Center (MTRC), School of Medicine, Kermanshah University of Medical Sciences, Kermanshah, Iran; ^4^Department of Cellular and Molecular Medicine, The Panum Institute, Faculty of Health and Medical Sciences, University of Copenhagen, Copenhagen, Denmark; ^5^Physical Medicine and Rehabilitation Research Center, Aging Research Institute, Tabriz University of Medical Sciences, Tabriz, Iran; ^6^Instituto de Investigación Sanitaria de Santiago (IDIS), Complejo Hospitalario Universitario de Santiago (CHUS), Servicio Gallego de Salud (SERGAS), Santiago de Compostela, Spain; ^7^Research Center for Molecular Medicine, Hamadan University of Medical Sciences, Hamadan, Iran; ^8^Translational Type 1 Diabetes Research, Department of Clinical, Research, Steno Diabetes Center Copenhagen, Gentofte, Denmark; ^9^Interventional Regenerative Medicine and Imaging Laboratory, Department of Radiology, Stanford University School of Medicine, Palo Alto, CA, United States

**Keywords:** extracellular vesicle, type 1 diabetes, exosomes, β-cell, immunomodulation, therapy

## Abstract

Type 1 diabetes (T1D) is a chronic disorder characterized by immune-mediated destruction of pancreatic insulin-producing β-cells. The primary treatment for T1D is multiple daily insulin injections to control blood sugar levels. Cell-free delivery packets with therapeutic properties, extracellular vesicles (EVs), mainly from stem cells, have recently gained considerable attention for disease treatments. EVs provide a great potential to treat T1D ascribed to their regenerative, anti-inflammatory, and immunomodulatory effects. Here, we summarize the latest EV applications for T1D treatment and highlight opportunities for further investigation.

## 1 Introduction

Type 1 diabetes (T1D) is caused by immune-mediated destruction of the insulin-producing β-cells resulting in a life-long insulin dependence (1). T1D can occur at any age but mostly arises already at a young age (2). The incidence of T1D has increased through the past three decades worldwide (3).

To establish stable glycemic control, patients with T1D need multiple daily insulin (MDI) injections or continuous insulin infusion through a pump (4). Despite technological advances, unstable glycemic control in patients continues to be a risk factor for diabetes-related metabolic and vascular complications (5, 6). Therefore, novel interventions are needed for the T1D treatment. Protecting and regenerating β-cell mass and improving insulin-producing capacity should be the primary purposes of any in-development and future T1D treatments.

Surgical islet transplantation and/or whole pancreas transplantation are the only current treatments for restoring the β-cells (7). Islet transplantation faces a limited source of islet donors and immune destruction after transplantation, requiring immunosuppression therapy (8, 9).

Ascribed to the autoimmune nature of T1D, immunotherapies aiming at the suppression/regulation of T cells or proinflammatory cytokines have also been developed over the past three decades (1). These treatments remain in active development; however, they have been only partly successful. Technological shortcomings, the limited translational success of adapting preclinical rodent studies to humans, and the ambiguity of the T1D pathogenesis have been involved in the current limitations of immunotherapies for T1D (5, 10, 11). Stem cell-based therapies that aim to replace or regenerate destructed β-cell are broadly investigated treatments (7). Although showing promising results, stem cell-derived β-cells need to be protected from immune-mediated destruction. One way to overcome this is by combining replacement therapy with immunomodulatory treatments (5).

One way to overcome the inflammatory condition and suppress the immune cells is using extracellular vesicles (EVs). EVs are therapeutic agents recently introduced as a new treatment for several autoimmune diseases, especially multiple sclerosis (MS), rheumatoid arthritis (RA), and T1D, with considerable immunomodulatory and regenerative effects (12–17). This review brings together current state-of-the-art advances in applying EVs as a treatment for T1D.

## 2 Extracellular Vesicles

### 2.1 Classification and Characterization

Nearly all cells can produce and release endosome-derived vesicles (30–2000 nm) called EVs (18, 19). EVs are important intercellular communication mediators and contain lipids, metabolites, DNA, proteins, and RNA species, such as miRNA, mRNA, tRNAs (20, 21). There are three main subtypes of EVs based on their mechanism of biogenesis: exosomes (EXOs), microvesicles (MVs), and apoptotic bodies (19). EXOs range from 30 to 180nm in size and are characterized by markers including tetraspanins (CD9, CD63, CD81, TSPAN29, and TSPAN30), ESCRT components, HSP70, ALIX, flotillin, MFGE8, and TSG101 (18, 19). EXOs components mainly include ESCRT-related proteins, microRNAs (miRNAs), messenger RNAs (mRNA) and other non-coding RNAs, cytoplasmic and membrane proteins, including receptors and major histocompatibility complex (MHC) molecules (18, 19, 22). EXOs arise from a complex multi-step process in which the plasma membrane is internalized to form a primary endosome, followed by intra-luminal vesicles (ILVs) formation within the endosome. At this stage, specific substrates enter the ILVs *via* ESCRTs or ESCRT-independent machines. The primary endosome is now considered a mature multivesicular body (MVB), including several ILVs. The MVB attaches to the plasma membrane and releases ILVs into the extracellular space called ‘EXOs’.

**Figure 1:**
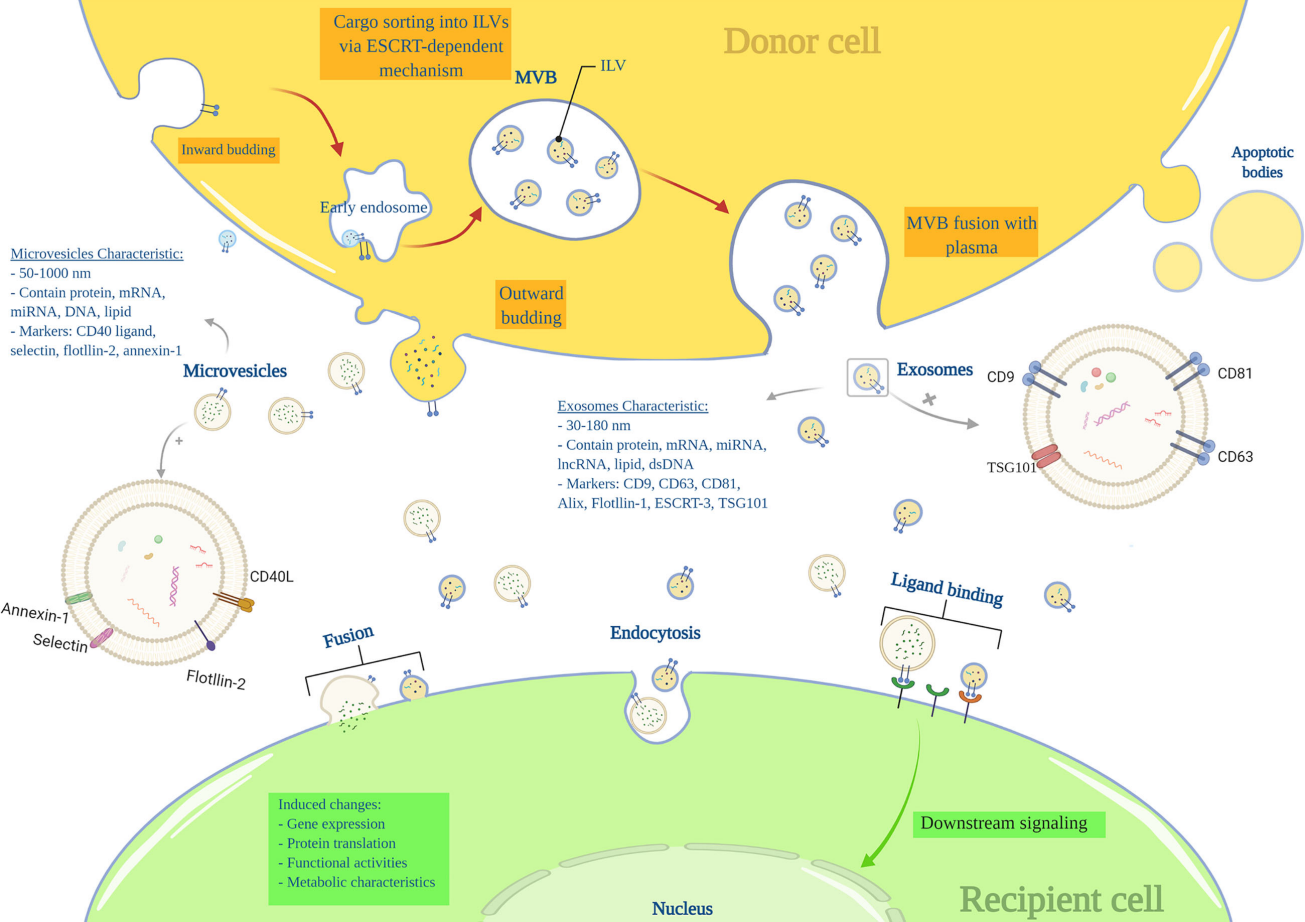
Extracellular vesicles characteristics and biogenesis.

MVs’ (50 to 1000nm) markers include integrins, selectins, and CD40 ligand, and their components are mRNA, miRNA, non-coding RNAs, and cytoplasmic and membrane proteins. MVs are generated by the outward budding of the plasma membrane (18, 23) (**Figure 1**). The apoptotic bodies (500 to 2000nm) are released from apoptotic cells and contain nuclear fractions and cell organelles (18, 24, 25).

Despite recent advances in the understanding of EV, in several published articles, the term “exosome” and “microvesicle” have been used interchangeably due to a lack of a uniform protocol for EV purification and incomplete knowledge of the EVs characteristics (18); as differences in the currently known characteristics such as size, density, and protein markers seem insufficient for a satisfiable distinction between EXOs and MVs (20, 26).

### 2.2 Interaction With the Target Cell and Biological Significance

EVs are naturally present in body fluids, including blood, urine, saliva, semen, sputum, and milk (21, 27, 28); and are released from many cells such as stem cells (29), progenitor cells (30), mesenchymal stromal cells (MSCs) (17), somatic cells like pancreatic beta cells (31), and many other cells.

EVs are recognized for their unique ability to transport different intact molecules between cells. Cell-to-cell communication mediated by EVs requires traveling and interacting with the plasma membrane. At the target site, EVs can act through membrane receptors and activate downstream intracellular signaling, or they directly fuse with the cell membrane and release their content into the target cell’s cytoplasm. EVs also can internalize by clathrin-mediated or clathrin-independent endocytosis (22, 32).

Several physiological functions have been identified for EVs in recent decades, as they play essential roles in the innate and acquired immune systems response (e.g., antigen presentation and activation of innate antiviral immune responses) (33–35). On the other hand, adverse roles have also been attributed to EVs when the condition is pathological, indicating that EVs are involved in developing or progressing many diseases, including type 1 and 2 diabetes, neurological diseases, autoimmune diseases, and cancers (e.g., primary and metastatic brain tumors, ovarian cancer, breast cancer, and pancreatic cancer) (15, 36–40).

In the last decades, EVs gained interest as a new treatment option in several diseases as they mediate intercellular signaling (20, 41). EV’s content profile and biological activity depend on their cell of origin and the microenvironment (42). They have shown great potential in inducing cell proliferation and tissue repair (43), angiogenesis (31), and improving some cellular functions (43). EVs have also shown considerable properties in modulating the immune system mainly by carrying immunomodulatory effectors, such as transcriptional factors (e.g., Nanog and Oct4), mRNAs, and cytokines (e.g., TGF-β and IL-10) (21, 44–46). For these reasons, diverse therapeutic properties made them popular, especially in drug delivery and regenerative medicine (41).

## 3 EVs for T1D

**Figure 2:**
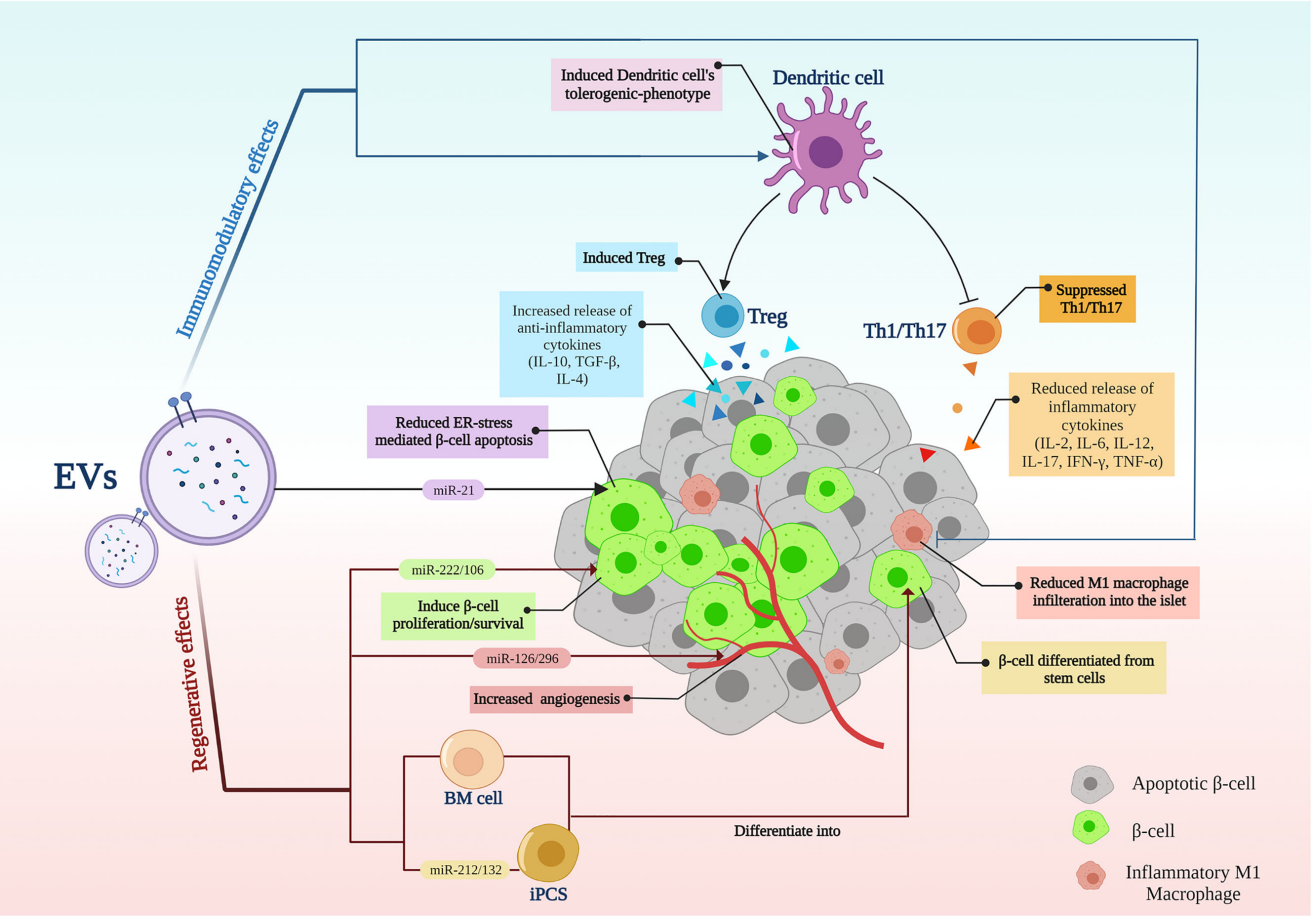
Extracellular vesicle application in type 1 diabetes; EVs modulate inflammation while recovering β-cells. EVs, extracellular vesicles; BM, Bone marrow; iPSCs, induced pluripotent stem cells; Th, T helper cell; Treg, regulatory T cell.

The main treatment strategies for T1D are immunotherapies (1) and cell replacement, including islet transplantation and stem cell differentiation into β-cells as a cell replacement method (7). As a new opportunity, EVs, with their therapeutic properties, had emerged (21, 42). EV’s simultaneous regenerative and immunomodulatory abilities align with new hypotheses about the T1D nature and the ideal treatments. According to the new speculations, an ideal treatment for T1D should be able to recover β-cells while modulating the immune system (10) (**Figure 2**).

### 3.1 Immunomodulatory Effects

T1D is essentially an autoimmune disease in which insulin-producing β-cells in the pancreas are invaded and destroyed by the immune system (1); Meanwhile, auto-reactive T cells have been identified as the primary attackers (47, 48). On the other hand, regulatory T cells have lost their efficiency in creating peripheral tolerance to β-cells (49). In T1D, EVs demonstrated immunomodulatory effects, mainly through suppressing reactive T cells and inducing regulatory T cells (**Table 1**).

**Table 1:** Immunomodulatory effects of extracellular vesicle application in type 1 diabetes.

Ev source	Ev concentration	Ev isolation method	Control	Experimental model	Administration route (*in vivo)*	Assay duration (*in vivo)*	Functional cargo	Downstream signaling	Downstream genes	Outcomes	Ref
BM-MSCs	*In vivo* (3 or 30 µg)	Column containing anion exchange resin	PBS, MSCs	*In vivo* (Adoptive transfer T1D scid-NOD mice model) In vitro (T cells, APCs)	IV tail vain	58 days	NA	NA	NA	Delayed onset of T1D, preserved islets, reduced insulitis and T cell infiltration, reduced APC and T cell activation, reduced Th1/Th17 population, reduced inflammatory cytokines (IL-17, IL-6, IFN-γ, TNF-a and IL-12) levels	(16)
β-cells (MIN6 cells)	*In vivo* (6×10^9^ particles per 0.2 ml PBS), *In vitro* (1.5×10^9^ or 4.5×10^9^ or 9×10^9^ particles/ml)	UC	PBS	*In vivo* (single injection of 140 or 200 mg/kg STZ-induced diabetic C57BL/ksJ), *In vitro* (INS-1 cells)	*In-situ* pancreas + IV tail vain	35 days	NA	NA	NA	Reduced number of pro-inflammatory macrophages in islets, increased islet angiogenesis	(31)
AD-MSCs	*In vivo* (50 µg exosomes in 1 ml PBS)	UC	NA	*In vivo* (5 injections of 50 mg/kg STZ-induced diabetic C57BL/6J mice)	Intraperitoneal	60 days	NA	NA	NA	Increased Treg numbers in spleen, increased immunomodulatory cytokines (IL-4, IL-10 and TGF-β) levels, reduced inflammatory cytokines (IFN-γ and IL-17) levels	(50)
Psh-Fas-anti-miR375 transfected hBMSCs + hBMSCs -PBMSCs co-culture	150 µg/ml protein concentration	Total exosome isolation reagent	hBMSC-exosome	*In Vivo* (single injection of 70 mg/kg STZ-induced diabetic humanized NSG mice) *In vitro* (0.2 mM STZ dissolved in 0.1 M citrate buffer-treated INS-1 cells, PBMCs)	IV	90 days	Anti-miR375-siFas, HGF	NA	Downregulation of Fas, miR-375 and miR-155 Upregulation of miR-let-7b and miR-let-7d	Reduced islet cells apoptosis, increased islet insulin release Increased Treg numbers, reduced inflammatory cytokines (IL-2 and IFN-γ) levels, reduced immune-rejection after islet transplantation	(51)
BM-MSC	6.25×10^7^ particles/ml	UC	MSC	*In vitro* (T cells)	NA	NA	NA	NA	Upregulation of IL-10 and IL-6 expression in DCs	Induced immature IL-10-secreting phenotype of DCs, increased IL-10 and IL-6 levels, increased Treg numbers, reduced Th17 numbers	(17)
CB-SC	80 µg exosomes in 2 ml PBS	UC	PBS	*In vitro* (PBMCs)	NA	NA	NA	NA	NA	Induced differentiation of monocytes into anti-inflammatory M2 macrophages	(29)

BM-MSCs, bone marrow-derived mesenchymal stromal cells; PBS, phosphate buffer saline; T1D, type 1 diabetes; SCID, severe-combined-immunodeficient; NOD, non-obese diabetic; IV, intravenous; AD-MSCs, adipose-derived mesenchymal stromal cells; APCs, antigen presenting cells; STZ, streptozotocin; DCs, dendritic cells; BM, bone marrow; hBMSCs, human bone marrow stromal cells; PBMSCs, peripheral blood mesenchymal stromal cells; CB-SC, cord blood stem cells; INS-1, rat insulinoma cells; NSG, NOD SCID gamma; HGF, hepatocyte-growth factor; UC, ultracentrifugation.

#### 3.1.1 Suppressing Auto-Reactive Immune Cells

T cells play a crucial role in the immune attacks on β-cells (47). CD4^+^ T cells are activated by β-cell antigens, which are presented by antigen-presenting cells (APCs), including macrophages and dendritic cells (DCs); these activated CD4^+^ T cells by producing cytokines, attract and induce the proliferation of CD8^+^ T and B cells in the islet, leading to insulitis (48). Insulitis indicates an immune attack on β-cells and is defined by the infiltration of inflammatory cells around and within the islets (52). Suppressing auto-reactive CD4^+^ T cells delays T1D onset/progression (1).

EVs have shown suppressive effects on auto-reactive T cells in T1D animal models, while MSC-derived EVs were shown to suppress the proliferation/activation of APCs, Th1 and Th17 cells *in vitro* and delayed T1D onset in mice (16). In line with these findings, releasing inflammatory cytokines by reactive CD4^+^ T cells including IFN‐γ, IL-12, TNF-α, IL-6, and IL-17 were reduced following EV treatment. The authors suggested that MSC-EVs might induce IL-10-secreting regulatory DCs; thereby, DCs subsequently suppressed Th1 and Th17 cells development (16). In another study, DCs preconditioned with MSC-derived EXOs represented an immature IL-10-secreting phenotype associated with reduced co-stimulatory molecules expression, which could reduce Th17 numbers in the islet-antigen-stimulated T cells *in vitro* (17). Therefore, Th17 cells are believed to contribute to the pathogenesis of autoimmune diseases; however, their role in T1D is not fully understood (53)

In addition to T cells, macrophages also infiltrate the pancreatic islets in the early stages of T1D and induce an inflammatory response resulting in insulitis and β-cell death (48). EXOs isolated from β-cells showed lower proinflammatory macrophage infiltration into the pancreatic islets in diabetic mice (31). In a study by Hu *et al.*, EXOs isolated from cord blood-derived stem cells converted patients’ blood monocytes into M2 macrophages with anti-inflammatory properties *in vitro*. M2 macrophages, called “educated” immune cells, can be re-injected into the T1D patients’ blood, thus modulating the immune system (29).

#### 3.1.2 Inducing Regulatory T Cells

Regulatory T cells (Tregs) are essential in establishing peripheral immune tolerance (54). Defective Treg-mediated immune regulation has been shown in numerous autoimmune disorders, including T1D (55). At least a subset of individuals with T1D have reduced FOXP3^+^ Treg frequency and function (49). Tregs from patients with T1D showed to be less potent to regulate the proliferation of autologous effector T cells (56) and produce mainly proinflammatory cytokines (57). However, T1D patients are believed to benefit from reinforcement of Tregs, even individuals who do not have reduced Treg frequency or function (49). Accordingly, EVs have demonstrated the potential to induce Tregs. EXOs derived from adipose-MSCs have been shown to restrain the autoimmune response of the streptozotocin (STZ)-induced T1D mouse model (50). These EXOs led to an increase in the splenic production of anti‐inflammatory cytokines TGF‐β, IL‐4, and IL‐10, with a decrease in the production of proinflammatory cytokines IL‐17 and IFN‐γ, as the results of an increased splenic CD25^+^FOXP3^+^ Treg population (50). Wen *et al.* suggested that EXOs isolated from human bone marrow stromal cells (hBMSC) and peripheral blood mononuclear cell (PBMC) co-culture could suppress immune reaction by amplifying Treg function in a humanized NOD SCID gamma (NSG) mouse model (51). In another study, T1D patients-isolated DCs preconditioned with MSC-derived EXOs induced a higher proportion of FOXP3^+^ Tregs in the islet-antigen-stimulated T cell population *in vitro*. These T cells also exerted an increased secretion of anti-inflammatory IL-10, TGF-β, and IL-6 molecules (17).

### 3.2 Inducing β-Cell Regeneration

Pancreatic β-cells regeneration is a promising strategy for T1D treatment. In this regard, EVs have shown considerable potential in inducing β-cell proliferation (**Table 2**). EXOs isolated from MSCs have been shown to induce islet cell regeneration and insulin secretion through upregulating pancreatic and duodenal homeobox1 (pdx1), TGF-β, and smad1/2 (12). Pdx1 is involved in β-cell differentiation, survival, and functional maintenance (62), and TGF-β is also responsible for cell proliferation and differentiation (63). Moreover, MSC-EXOs demonstrated higher regenerative potential when compared with parental MSCs (58). EXOs derived from menstrual blood-derived MSCs have also shown similar results in inducing β-cell regeneration through pdx1 upregulation in T1D rats (59). Healthy adipocyte-derived EVs also improved the survival, proliferation, and insulin-producing function of pancreatic β-cells (43). In a study by Tsukita *et al.*, bone marrow transplantation promoted β-cell regeneration in T1D mice by releasing EXOs containing miR-106b-5p and miR-222-3p into the bloodstream. These microRNAs encapsulated in EXOs reached β-cells and downregulated p21Cip1 and p27Kip1, which are negative controllers of β-cell regeneration after injury, inducing β-cell proliferation (60).

**Table 2:** Regenerative effects of extracellular vesicle application in type 1 diabetes.

ev source	Ev concentration	Ev isolation method	Control	Experimental model	Administration route (*in vivo)*	Assay duration (*in vivo)*	Functional cargo	Downstream signaling	Downstream genes	Outcomes	Ref
BM-MSCs	*In vivo* (200 µg/ml vehicle)	UC	BM-MSCs	*In vivo* (single injection of 50 mg/kg STZ-induced diabetic albino rats)	Intraperitoneal	28 days	NA	NA	Upregulating of PDX1, TGF-β, smad2/3 and insulin	Induced β-cell proliferation and insulin secretion	(12)
BM-MSCs	*In vivo* (0.4 µg/ml to final volume of 100 µL)	UC	BM-MSCs Vitamin-D	*In vivo* (single injection of 55 mg/kg STZ-induced diabetic albino rats)	IV tail vain	90 days	NA	NA	Upregulation of PDX1, smad2/3, PAX4, nueroD and insulin	Induced higher levels of β-cell proliferation and insulin secretion than MSCs	(58)
Menstrual blood-derived MSCs	*In vivo* (10 µg exosomes dissolved in PBS)	Exosome isolation kit	Menstrual blood-derived MSCs, PBS	*In vivo* (single injection of 60 mg/kg STZ-induced diabetic Wistar rats)	IV tail vain	40 days	NA	NA	Upregulation of PDX1	Induced β-cell proliferation and insulin secretion	(59)
Adipocytes (Healthy donor)	*In vitro* (5×103 or 10×103 EVs/target cell, 1×108 EVs/islet)	UC	Inflamed adipocyte-derived EVs, subcutaneous adipose tissue-derived EVs	*In vitro* (INS-1 cells, EndoC-βH3 β cells)	NA	NA	NA	GSK-3β, MAPK/ERK1/2 and PERK activation	Upregulating of eIF2a, adiponectin, PDX1 and NKX6.1	Improved β-cell survival, proliferation and insulin-producing function	(43)
BM-cells	NA	ExoQuick-TC	NA	*In vivo* (5 injections of 50 mg/kg STZ-induced diabetic C57BL/6J mice)	NA	50 days	miR-222-3p miR-106-5p	NA	Downregulation of p21Cip1 and p27Kip1	Induced β-cell proliferation	(60)
PCs	*In vivo* (100 ng in PBS to final volume of 300 µL)	UC	PCs PBS	*In vivo* (single injection of 150 mg/kg STZ-induced diabetic C57BL/6J mice)	IV tail vain	28 days	NA	NA	NA	β-cell regeneration	(61)

BM-MSCs, bone marrow-derived mesenchymal stromal cells; PBS, phosphate buffer saline; IV, intravenous; STZ, streptozotocin; PCs, pathfinder cells; INS-1, rat insulinoma cells; EVs, extracellular vesicles; UC, ultracentrifugation.

Pathfinder cells (PC) (pancreas-derived progenitor cells) were recently introduced as a novel cell type that can be isolated from the adult rat pancreatic ducts and induced to form islet-like structures *in vitro* (64). PCs have been shown to stimulate regeneration of damaged mice pancreatic islets (65). In order to extend the latter study, McGuinness *et al.* suggested that PC-derived MVs (not EXOs) induce islet regeneration and restore their function in the STZ diabetic mouse model. The results of the PC-MVs were similar to the PC treatment (61), which demonstrated that PCs in the Stevenson *et al.* study (65) possibly acted through releasing MVs.

### 3.3 Improving Islet Transplantation Outcome

Destruction of transplanted islets due to immune rejection and poor vascularity limits the broad application of islet transplantation in T1D patients (66). Therefore, improving transplanted islet cell survival by protecting these islets against the immune system and enhancing angiogenesis within and/or around them seem beneficial and essential. In this context, EVs showed promising effects (**Table 3**).

**Table 3:** Extracellular Vesicle application after islet transplantation in type 1 diabetes.

Ev source	Ev concentration	Ev isolation method	Control	Experimental model	Administration route (*in vivo)*	Assay duration (*in vivo)*	Functional cargo	Downstream signaling	Downstream genes	Outcomes	Ref
EPCs	10 µg/ml MVs	UC	Vehicle, MVs pretreated with RNase	*In vivo* (SCID mice) *In vitro* (human islets, human IECs)	Loaded by a matrigel (SC implantation)	7 days	miR-126 and miR-296	PI3K/Akt/eNOS activation	NA	Induced proliferation and migration in IECs, increased neoangiogenesis in transplanted islets and improved islets viability and function	(30)
β-cells	15 µg/ml	Size exclusion chromatography	PBS	*In vivo* (SCID mice for iPSCs-derived β-cells transplantation) *In vitro* (iPSCs)	NA	28 days	miR-212 and miR-132	NA	Downregulation of Nanog, oct4 and FBW4 Upregulation of FoxA2, PDX1, NGN3, NKX6.1, NKX2.5 and insulin	Induced differentiation of iPSCs into β-cells capable of releasing insulin in response to glucose, reducing blood glucose levels in diabetic mice	(67)
UC-MSCs	*In vivo* (7.05×10^10^ ± 3.69×10^10^ exosomes in 1 ml PBS) *In vitro* (200 µg/ml)	UC	Non-encapsulated exosomes	*In vivo* (single injection of 180 mg/Kg STZ-induced diabetic C57BL/6J mice) *In vitro* (PBMCs, activated RAW264.7 macrophages, splenocytes)	Loaded by hybrid Alginate microcapsule	167 days	NA	NF-kB inhibition	Downregulation of G-CSF, IFN-γ, LIF, kc, MIP-2, IL-6, VEGF	Reduced FBR (inflammation and fibrosis) to transplanted islets, improved transplanted islets viability and	(68)
β-cells	*In vivo* (10 µg/each injection) *In vitro* (1 µg protein of EMNVs)	UC	NI3H3T3 cells- EMNVs, PBS	*In Vivo* (single injection of 140 mg/kg STZ-induced diabetic NSG mice) *In vitro* (BM cells)	Loaded by matrigel containing BM cells (SC implantation)	60 days	NA	NA	Upregulation of FoxA2, PDX1, MafA and insulin	Induced BM cells differentiation into functional insulin-producing β-cells and controlled blood glucose levels in diabetic	(69)
hUC-MSC	50 µg/ml	UC	NA	*In vitro* (β-cells under hypoxic condition)	NA	NA	miR-21	Inhibiting p38 MAPK pathway	Downregulation of ER stress-related proteins elF-2a, CHOP, GRP78, GRP94	Reduced hypoxia-mediated apoptosis of β-cells, reduced ER-stress, increased islet survival after transplantation	(70)
hUC-MSC	0.5, 10 and 20 µg/ml	UC	Exosome-free conditioned media	*In vitro* (porcine islets under hypoxic condition)	NA	NA	NA	NA	Upregulation of HIF-1a, VEGF and PDH2	Reduced hypoxia-mediated cell death and dysfunction of β-cells	(71)
hUC-MSC	40 µg/ml	UC	Exosome-free conditioned media, MSCs	*In vitro* (mouse islets under hypoxic condition)	NA	NA	VEGFA (mRNA and protein)	PI3K	Upregulation of VEGF and Bcl-2 Downregulation of BAD and BAX	Reduced hypoxia-mediated cell death and dysfunction of β-cells	(72)

EPCs, endothelial progenitor cells; MVs, microvesicles; PBS, phosphate buffer saline; T1D, type 1 diabetes; SCID, severe-combined-immunodeficient; IECs, islet endothelial cell line; SC, subcutaneous; iPSCs, induced pluripotent stem cells; UC-MSCs, umbilical cord mesenchymal stem cells; PBMCs, peripheral blood mononuclear cells; non-obese diabetic; FBR, foreign body response; BM ,bone marrow; EMNVs, extracellular-mimetic nanovesicles; APCs, antigen presenting cells; STZ, streptozotocin; ER, endoplasmic reticulum; hUC, human umbilical cord; GRP, glucose-regulated protein; UC, ultracentrifugation.

hBMSCs derived EXOs suppressed transplanted islet apoptosis and improved their function in a humanized NSG mouse model by co-delivering short interfering RNA (siRNA) against Fas receptor and miR-375 inhibitor (51). Evidence suggests that Fas activity and miR-375 impair islet cell proliferation and insulin secretion (73–75).

Foreign body response (FBR) is an inflammatory reaction to the transplantation, leading to dense fibrotic tissue forming around the microcapsules-containing islets, restricting the survival and insulin-producing function of islets in rodent models (76, 77). Xenotransplantation of rat islets encapsulated in hybrid alginate microcapsule-loaded by EXOs derived from human umbilical cord MSCs (UC-MSCs) attenuated the immune-based FBR and islet-engulfed fibrosis after transplantation. It increased the survival of transplanted islets in an immunocompetent mouse model of T1D. These EXOs suppressed inflammatory macrophage and T cell activation/proliferation followed by a reduced production of inflammatory cytokines, including MCP-1, IL-2, IL-6, IL-12, IL-22, and TNF-α (68).

Transplanted islets often experience hypoxia due to poor vascularity (78). Hypoxia causes β-cells death and plays a crucial role in destroying the transplanted islets (79). EXOs derived from human UC-MSCs have been demonstrated to improve β-cell survival under a hypoxic condition by delivering miR-21, resulting in reduced endoplasmic reticulum (ER) stress and p38 MAPK suppression (70). As ER stress activates p38-dependent apoptosis (80), ER stress reduction leads to a decrease in β-cell apoptosis (70). EXOs derived from human UC-MSC were also able to protect the survival and function of porcine islets from hypoxia by inducing hypoxia-inducible factor 1α (HIF-1α) expression (71). In another similar study, Keshtkar et al. also observed similar results for UC-MSC-derived EXOs in improving the survival and function of mouse islets; but this time, they introduced VEGF as the leading player in these results. They showed that UC-MSC-derived EXOs contained VEGF (mRNA and protein) and increased VEGF expression in islet cells (72). VEGF can enhance the viability and function of transplanted islets in the early days after transplantation, and this effect of VEGF is independent of neovascularization since neovascularization in transplanted islets occurs days to weeks later (81–83).

EVs also demonstrated potential in improving islet revascularization after transplantation. MVs derived from endothelial progenitor cells (EPCs) induced angiogenic behavior in islet endothelium *in vitro* and enhanced vascularization of transplanted islets in severe combined immunodeficient (SCID) mice. This proangiogenic effect was acquired by delivering miR-126 and miR-296 (known as proangiogenic microRNAs) and activating the PI3K-Akt and eNOS signaling pathways further. Indeed, EPC-derived MVs improved insulin secretion and survival of the transplanted islets (30). Moreover, MVs isolated from the β-cell line preserved islet cells’ function by inducing islet neovascularization in streptozotocin-diabetic mice (31).

Another problem in islet transplantation is limited sources of functional islets for transplantation. Therefore, several studies have developed alternative insulin-producing cells by inducing differentiation of stem/progenitor cells (84). In this regard, applying EV-mimetic nanovesicles (EVMNs) derived from a pancreatic β-cell line to a subcutaneous matrigel platform containing bone marrow cells in diabetic NSG mice induced differentiation of islet-like clusters of insulin-producing cells with capillary networks from bone marrow cells. These islet-like insulin-producing cells could control the blood glucose levels over 60 days (69).

Bai et al. used β cell-derived EVs to induce β-cell differentiation from induced pluripotent stem cells (iPSCs) and suggested the critical role of miR-212/132 (encapsulated in these EVs) in this process, which inhibited FBW7 to reinstate neurogenin 3 (NGN3) expression. In the following, NGN3 is bound to PDX1 to induce endogenous miR-212/132 expression resulting in the insulin-producing function of β-cells (67). NGN3 is a transcription factor involved in pancreas development (85, 86), which induces differentiation of islet cell precursors, and FBW7 is a ubiquitin ligase that has a negative impact on the NGN3 stability (87).

## 4 Future Considerations

While it is widely believed that T1D is caused by immune system dysregulation and autoimmunity, another hypothesis has been put forward stating that the pathogenesis of T1D is triggered *via* β-cell itself and consequently causes an unregulated autoimmune response (10), a phenomenon entitled “un-masked β-cell” (5). This might partially explain why immunotherapies alone are not satisfactory. One possible cause of the “un-masked β-cell” is β-cell stress. Stressed β-cells through HLA-I and chemokine CXCL10 secretion hyper-expression attract leukocytes to the islet and precede insulitis. Therefore, reducing this cellular stress in β-cells may effectively mitigate this phenomenon (10, 88). As mentioned, EVs can efficiently reduce β-cell stress (70). The “un-masked β-cell” hypothesis supports therapies that help β-cell recovery/regeneration while modulating the immune system. Since immune system dysregulation is thought to result from an initial β-cells misbehave; thus, the consequent autoimmunity cannot be resolved before β-cell recovery (10). In this regard, EV therapy suggests both effects.

Accordingly, one practical approach for T1D would be inducing selective immune tolerance to β-cell autoantigens (10). Nanoparticles and microparticles have been used as delivery packets for autoantigens such as proinsulin or GAD65 to induce a tolerogenic phenotype in DCs, characterized by the reduced ability of DCs to activate inflammatory auto-reactive T cells resulting in increased differentiation of FOXP3^+^ Treg cells (89). In this regard, EV, as a drug delivery tool (18), can also induce immune tolerance in DCs *via* delivering β-cell autoantigens. Indeed, MSC-derived EVs were shown to induce a tolerogenic phenotype in DCs resulting in anti-islet T cells reduction, although not by β-cell autoantigen transfer (16, 17).

Although knowledge on EV therapy for T1D is not yet leading to a conclusive outcome, based on the studies we reviewed here, the most prominent cell sources for EV isolation for therapeutic applications in T1D include BMSCs, β-cells, and UC-MSCs. When it comes to inducing proliferation and improving the insulin production/secretion function of β-cells, BMSCs-derived EVs showed considerable potency (12, 58, 60). Moreover, when it comes to inducing differentiation of β-cells from pluripotent cells, β-cells-derived EVs are the most used (67, 69). EVs derived from UC-MSCs were also reported to protect β-cells after transplantation with high efficiency (70–72).

However, interpretation of the results of current studies should be made with care; since most studies did not adequately address the potential effects associated with EVs co-isolates (22); therefore, it is possible that some of the obtained results were affected or caused by the co-isolates. In addition, there are still many other considerations associated with EV isolation, evaluation, and functional assays, which are not adequately addressed in most studies. EV isolation and concentration methods, including ultracentrifugation, ultrafiltration, density gradients, precipitation, size exclusion chromatography, and immuno-isolation, cannot effectively isolate various EV subtypes (including EXOs and MVs) and are partially successful in specificity to EV isolation (6, 22). In addition, there is still no standard way to evaluate the stability (90) and functionality of these isolated EVs, so it is not clear how many EVs are ultimately involved in the reported results.

Clinical application of EVs for T1D has been limited so far. This could be partially because much preparation is needed to bring EV therapy into the clinical practice; upgrading and standardizing EV isolation/purification methods, obtaining the most efficient dose and the number of EV injections, producing EV on a large scale, and ensuring EV safety are some of the most prominent issues that must be addressed before the entrance of EV to the clinics (6). To study the effect of EV therapy on β-cell mass and glycemic control, one study at phase 1 was conducted in 2014, where cord blood MSC-derived EVs were used to treat twenty T1D patients (NCT02138331). However, this study seems never to be completed, and the data is not published. This is the only clinical trial performed in T1D using EVs to the best of our knowledge. Obviously, more clinical trials are needed to evaluate EV effectiveness for T1D.

## 5 Conclusion

Here we reviewed the studies that used EVs for T1D treatment in pre-clinical settings and further highlighted the lack of clinical application knowledge in the field. As an effective treatment for T1D, EVs are thought to regulate the immune system at innate and adaptive levels, possibly by inducing tolerance in DCs to β-cell autoantigens. Based on the recent speculation about the pathogenesis of T1D, which emphasizes the β-cell as a key player in initiating autoimmunity, EV therapy could continue to present itself as an effective and scalable treatment option. It was “postulated” that EVs can reduce cellular stress in β-cells and, therefore, reduce the immune system attack. By inducing proliferation and regeneration in β-cells, EVs also restore destructed islet mass. Moreover, they can increase the survival rate of transplanted islets by diminishing immune rejection and by enhancing angiogenesis.

In a nutshell, studies reviewed here have provided excellent guidelines to use EVs features to design specific clinical trials that utterly could generate an alternative to standard treatment. However, EVs continue to present some practical challenges for their use as a clinical-grade therapy, which must be addressed in the future.

## Author Contributions 

All authors listed have made a substantial, direct, and intellectual contribution to the work, and approved it for publication.

## Funding

The Lundbeck foundation grant R303-2018-3148 supported this work.

## Conflict of Interest

The authors declare that the research was conducted in the absence of any commercial or financial relationships that could be construed as a potential conflict of interest.

## Publisher’s Note

All claims expressed in this article are solely those of the authors and do not necessarily represent those of their affiliated organizations, or those of the publisher, the editors and the reviewers. Any product that may be evaluated in this article, or claim that may be made by its manufacturer, is not guaranteed or endorsed by the publisher.
